# Bhattacharyya Parameter of Monomial Codes for the Binary Erasure Channel: From Pointwise to Average Reliability

**DOI:** 10.3390/s21092976

**Published:** 2021-04-23

**Authors:** Vlad-Florin Drăgoi, Gabriela Cristescu

**Affiliations:** 1 Faculty of Exact Sciences, Aurel Vlaicu University of Arad, 2 Elena Dragoi Street, 310130 Arad, Romania; gabriela.cristescu@uav.ro; 2 LITIS, University of Rouen Normandie, Avenue de l’Université, 76801 Saint-Etienne-du-Rouvray, France

**Keywords:** Bhattacharyya parameter, average reliability, monomial code, polar code, order relation, binary erasure channel

## Abstract

Monomial codes were recently equipped with partial order relations, a fact that allowed researchers to discover structural properties and efficient algorithm for constructing polar codes. Here, we refine the existing order relations in the particular case of the binary erasure channel. The new order relation takes us closer to the ultimate order relation induced by the pointwise evaluation of the Bhattacharyya parameter of the synthetic channels, which is still a partial order relation. To overcome this issue, we appeal to a related technique from network theory. Reliability network theory was recently used in the context of polar coding and more generally in connection with decreasing monomial codes. In this article, we investigate how the concept of average reliability is applied for polar codes designed for the binary erasure channel. Instead of minimizing the error probability of the synthetic channels, for a particular value of the erasure parameter *p*, our codes minimize the average error probability of the synthetic channels. By means of basic network theory results, we determine a closed formula for the average reliability of a particular synthetic channel, that recently gain the attention of researchers.

## 1. Introduction

One of the most striking developments in coding theory in the last two decades is probably the theory around polar codes. In his seminal article [1], Arikan demonstrated, for the first time, that one could achieve the capacity of binary discrete memoryless channels (BDMC), using both efficient encoding and efficient decoding algorithms. The so-called polar codes are now present in the fifth generation (5G) technology [2]. Indeed, polar code was elected as the standard coding technique for the control channel in support of the enhanced mobile broadband service, one of the major parts in 5G wireless network technology. Getting back to the three principal directions on which coding theory evolved, polar coding seemed to be unrelated to classical algebraic coding. Typically, the construction of polar codes does not come from any particular structure in the code, but rather from the process of channel polarization. However, polar codes are closely related to Reed–Muller codes, as pointed out even by Arikan [1]. Hence, polar and Reed-Muller code share a common algebraic description [3,4]. More precisely, they are sub-classes of a larger family of algebraic codes called decreasing monomial codes (DMC). The structure underlying DMCs and its algebraic formalism was applied in conjunction with other fields, e.g., in the context of quantum error correcting codes [5,6,7], post-quantum cryptography [8,9,10] and network reliability [11,12,13].

Several challenges regarding polar coding, among which the efficient construction of polar codes given a specific BDMC, were proposed. Arikan’s initial technique [1] was improved by several authors [14,15,16,17,18,19,20,21,22]. Let *W* denote a BDMC, *m* a fixed integer and u a binary vector of length m. The main idea of the construction of polar codes is to estimate the reliability of the synthetic channels $\left\{W^{u}\;|\;u\in {\left\{0,1\right\}}^{m}\right\}$. For that, one might use the Bhattacharyya parameter $B\left(W^{u}\right)\left(p\right)$, where *p* denotes the error probability of the channel *W*. The message parts of a polar code of length $2^{m}$ and dimension *k* are allocated to the *k* sub-channels $W^{u}$ having the smallest $B\left(W^{u}\right)$. Hence, one might classify the set of $W^{u}$ into “good” (reliable) or “bad” (non-reliable). For a fixed value of *p*, the values $B\left(W^{u}\right)\left(p\right)$ are put in order. In other words, when the parameter *p* is fixed, any distinct pair of channels $W^{u},W^{v}$ satisfies either $B\left(W^{u}\right)\left(p\right)\leq B\left(W^{v}\right)\left(p\right)$ or $B\left(W^{v}\right)\left(p\right)\leq B\left(W^{u}\right)\left(p\right).$ In this case, we say that a channel $W^{u}$ is point-wise more reliable than a channel $W^{v}.$ However, when considering the whole interval p∈[0,1], ranking the synthetic channels becomes complicated. In this case, we say that $W^{u}$ is globally more reliable than $W^{v}$, and write u≤v, if and only if $\forall p\in \left[0,1\right]\;,\;B\left(W^{u}\right)\left(p\right)\leq B\left(W^{v}\right)\left(p\right).$

Estimating how reliable a synthetic channel is can be done in several ways, Monte Carlo simulations being among the most common ones. Arikan, in his seminal paper [1], proposed such a method for determining the error probability of each synthetic channel which yields a relatively efficient generating algorithm. It could be possible to employ recent development in Monte Carlo such as those in [23,24] in order to improve Arikan’s idea. However, the most efficient techniques for constructing polar codes are exploiting order relations between the synthetic channels. One of the most efficient techniques that orders the set of synthetic channels (with respect to the concept of globally more reliable), provides sub-linear complexity construction [14]. It exploits the existence of a partial order (denoted by ⪯) on the set of synthetic channels [4]. This partial order is compatible with the notion of being globally more reliable, i.e., u⪯v⇒u≤v. Even though ⪯ provided a contribution to understanding polar codes, i.e., their structure and construction, simulations show that ⪯ is far from ordering $B\left(W^{u}\right)$ optimally. Hence, in a recent article, ⪯ was refined [25]. Compared to Monte Carlo methods, the techniques based on order relations valid for polar codes require a small number of computations, only for a fraction of the synthetic channels, as they exploit the rules induced by the order relations.

In the analysis of the performance of several families of codes, among which are polar, Reed–Muller, cyclic and BCH codes, the communication channel that received a lot of attention is the binary erasure channel (BEC). When polar codes are designed for BEC(p) (in this particular case, *p* denotes the erasure probability), all the synthetic channels $\left\{W^{u}\;|\;u\in {\left\{0,1\right\}}^{m}\right\}$ are also BEC. In this case, the erasure probability of $W^{u}$ is equal to the Bhattacharyya parameter of $W^{u}.$ Here, we analyze this particular channel. Our choice is motivated by several results and methods. First of all, the simplicity of this channel makes the theoretical proofs significantly simpler and easier. Moreover, many of the properties that hold for the BEC turn out to be valid for more general channel models. For example, the proof of Reed–Muller codes achieving the capacity of a communication channel started with the BEC [26,27]. Codes that admit a doubly-transitive automorphism group or having large orbits under the action of their permutation group achieve the capacity of the BEC [27,28]. In [29], the authors analyze threshold points for $W^{u}$ in the case of BEC, a fact that allows them to propose sets of asymptotically “good” channels. Recently, in [30] the authors analyzed the Bhattacharyya parameter of polar codes for the BEC using network reliability theory. They have proposed simple approximations of $B\left(W^{u}\right)$. These were used to determine sub-intervals of [0,1], where polar codes coincide with Reed–Muller codes. They have also managed to determine new sets of asymptotically “good” channels.

### 1.1. Polar Codes Are Strongly Decreasing Monomial Codes

Polar codes over the BEC satisfy an order relation that is finer than ⪯.. Hence, we define another order relation ${⪯}_{d}$ on the set of monomials on *m* variables $M_{m}=\left\{1,x_{0},\cdots ,x_{m-1},x_{0}x_{1},\cdots ,x_{0}x_{1}\cdots x_{m-1}\right\}$, coming closer to the ≤ relation, i.e., we have

$$\forall f,g\in M_{m}\;f⪯g\Rightarrow f{⪯}_{d}g\Rightarrow f\leq g.$$

The relation ${⪯}_{d}$ allows one to compare monomials with equal degrees that were not comparable with respect to ⪯, e.g., $x_{1}x_{2}{⪯}_{d}x_{0}x_{3}.$ The idea of ${⪯}_{d}$ came from the link between the set of monomials of degree *d* in $M_{m}$ and the set of partitions/Young diagrams inside the d×(m−d) grid (see Proposition 3.7.8 in [3]). From that, looking at order relations on partitions came as a natural idea, and the most common one is the dominance order [31]. The order ${⪯}_{d}$ is exactly defined as the dominance order on partitions inside a fixed grid.

The main result in this section can be stated as follows

**Theorem** **1.** *Polar codes over the binary erasure channel are strongly decreasing monomial codes.*

In the proof of this theorem, we will need to demonstrate two useful properties of this new order

- given two monomials in $f,g\in M_{m}$ such that $f{⪯}_{d}g$, then, for any multiples fh,gh with gcd(h,f)=1 and gcd(g,h)=1 we have $fh{⪯}_{d}gh,$ where gcd(f,g) denotes the greatest common divisor of f,g.
- two particular monomials are the key ingredients in the proof, $x_{1}x_{2}{⪯}_{d}x_{0}x_{3}.$ We show that for all $p\in \left[0,1\right],B\left(W_{}^{x_{1}x_{2}}\right)\left(p\right)\leq B\left(W_{}^{x_{0}x_{3}}\right)\left(p\right)$, and in general that any pair of monomials of degree 2 f,g, satisfying that $f{⪯}_{d}g$ has the property for all $p\in \left[0,1\right],B\left(W^{f}\right)\left(p\right)\leq B\left(W^{g}\right)\left(p\right).$

**Figure 1:**
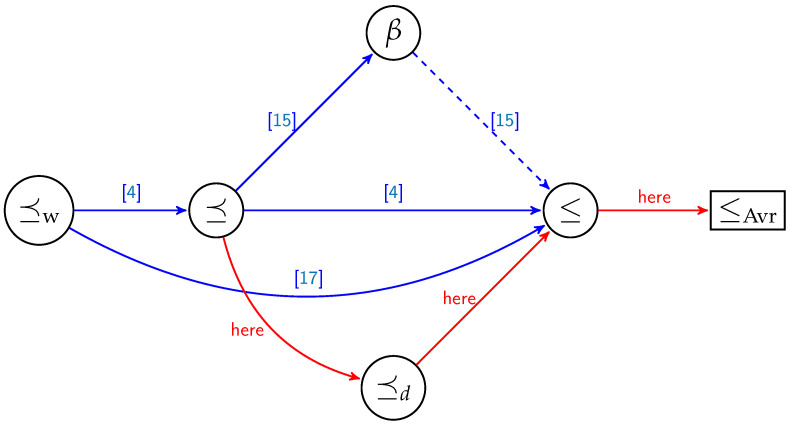
Order (${⪯}_{w},⪯,{⪯}_{d},\leq$) and preorder relations ($\leq_{\mathrm{Avr}}$) for monomials codes over the BEC. The connections in red are the results coming from this article. The dotted edge from β to ≤ represents an order relation that is valid only for a sub-interval of [0,1].

Even though ${⪯}_{d}$ gets us closer to the ultimate order relation ≤, we know that ${⪯}_{d}$ is a partial order relation. ${⪯}_{d}$ seems to perform as well as the order relation from [25], being much simpler to describe and analyze than the order in [25]. Furthermore, in [25] the authors determine new order relations based on some hypotheses which are not algebraically easy to express, and which are to be tested each time we change the parameters of the code. Figure 1 illustrates how our results fit into state-of-the-art order relations in conjunction with polar coding.

Now, as ${⪯}_{d}$ is thinner than ⪯, one could use it in order to determine new sets of comparable monomials and thus generate polar codes in a more efficient manner. Indeed, as more monomials are comparable with respect to ${⪯}_{d}$, they induce new chains in the poset $\{M_{m},{⪯}_{d}\}.$ This enables us to reduce the number of non comparable elements and also the number of strongly decreasing monomial codes compared to decreasing monomial codes. These two ideas are being illustrated as possible methods for reducing the complexity of the construction algorithm, hence directing towards possible practical applications.

### 1.2. Average Reliability of Synthetic Channels

**Figure 2:**
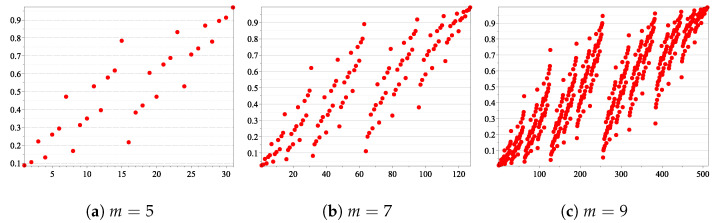
Average Bhattacharyya parameter. On the x-axis are the integer values of the binary vectors $u\in {\left\{0,1\right\}}^{m}$, and on the y-axis are the values Avr($B(W^{u})$).

Hence, we are still left with elements that are not comparable and for which we need to compute $B\left(W^{u}\right).$ In order to overcome this issue, we propose an alternative solution. Suppose that the erasure probability of the channel *p* changes with respect to the uniform distribution over the closed interval [0,1]. Instead of constructing, for each *p*, the corresponding polar code, we propose to construct the best polar code in average. More exactly, we consider the average reliability of the synthetic channels $W^{u}$, $\mathrm{Avr}\left(W^{u}\right)=\int_{0}^{1}B\left(W^{u}\right)dp$, and choose those u that minimize this quantity. As the average reliability induces a total order relation (see Figure 2), there is only one polar code for a given dimension and length. It is the linear code that minimizes the average error probability for all p∈[0,1]. Hence, it might be less efficient than polar codes designed for a particular value of *p*, but it has the best performance on average.

The preorder $u\leq_{\mathrm{Avr}}v\Leftrightarrow \mathrm{Avr}\left(W^{u}\right)\leq \mathrm{Avr}\left(W^{v}\right)$ induces a complementarity property with respect to the integral operator over [0,1], as defined in [32,33] in the case of two-terminal networks. We retrieve a similar property, i.e., $\mathrm{Avr}\left(W^{u}\right)=1-\mathrm{Avr}\left(W^{\bar{u}}\right),$ where $\bar{u}$ is the bit-wise complement of u, in the context of monomial codes. Our simulations have shown that, considering the relation $\leq_{\mathrm{Avr}}$ in the set of the synthetic channels, in each sub-interval (i/10,(i+1)/10), for 0≤i≤9, we have a rough proportion of $2^{m}/10$ binary vectors u. So, roughly speaking, a uniform distribution could be used to approximate the number of u inside each sub-interval, with respect to Avr. However, our result is not constructive, in the sense that it does not fully characterize exactly the u that belong to a specific interval. An answer to this question might provide an extremely efficient method for constructing polar codes and give much more insight into the synthetic channels $W^{u}.$

#### Threshold Points for Sharp Transitions

Determining the threshold point of $B\left(W^{u}\right)$ is in general a difficult task [29,34]. In [29], the authors analyze a particular synthetic channel $W^{\left(1^{i}0^{m-1}\right)}$, for which asymptotic threshold points were determined. The conditions on *i* and *m* were further improved in [25]. Based on some basic notions and facts from network theory, we determine an exact formula for the average reliability of $W^{\left(1^{i}0^{m-1}\right)}$. The main result is

**Theorem** **2.** *Let $u=(1^{i}0^{m-i}).$ Then*

(1) AvrWu=1−12i+2i−m2i

This allows us to determine the exact threshold point of this particular channel. Moreover, we demonstrate that for any $i\leq m-\log_{2}\left(m\right)-\log_{2}\left(\log_{2}\left(m\right)\right)$, the channel $W^{\left(1^{i}0^{m-i}\right)}$ has an average Bhattacharyya parameter that tends to zero when *m* goes to infinity, i.e., $W^{\left(1^{i}0^{m-i}\right)}$ is asymptotically “good” on average. Another consequence of our formula is that for any monomial $g{⪯}_{d}x_{m-i+1}\cdots x_{m}$ with $i\leq \log_{2}\left(\log_{2}\left(m\right)\right)$ is such that $\mathrm{Avr}(W^{g})$ tends to zero when *m* goes to infinity.

Another significant implication of our result is that any synthetic channel in the RM(i,m) is asymptotically “good” on average, for any $i\leq \log_{2}\left(\log_{2}\left(m\right)\right).$

### 1.3. Outline of the Article

The main concepts, notations and properties that are used all over the paper are introduced in Section 2. We introduce the background on coding, referring to the monomial codes in Section 2.1, the polar codes in Section 2.2 and to various manners of comparing them in Section 2.3. The two-terminal networks and their reliability are discussed in Section 2.4. The similarity between the behavior of the Bhattacharyya parameters and the reliability polynomial in assessing the monomial codes is emphasized in Section 2.5. The relationship between the order relations and the corresponding order structures over the set of polar codes over the binary erasure channel is studied in Section 3. The main result in this section proves that the polar codes over the binary erasure channel are strongly decreasing monomial codes. In the same section, two ideas pointing to possible practical applications of the order relation ${⪯}_{d}$ are developed. The concept of average reliability of a synthetic channel is introduced in Section 4. The properties of this operator are studied in Section 4.1 and the relation to the β-expansion is presented in Section 4.2. Finally, the threshold points of the binary erasure polarization sub-channels are determined in Section 4.3. Simulations and numerical examples are included to illustrate all the new results. We conclude our article in Section 5.

## 2. Background and Preliminary Results

Let us begin by listing some of the usual notations from coding theory that are going to be used in this article. $F_{2}$ will denote the finite field with two elements {0,1}. Let k,n be two strictly positive integers and k≤n. A code C of length *n* and dimension *k* is a vector sub-space of $F_{2}^{n}$ of dimension k. In this article, we focus out attention on a particular family or linear codes, namely monomial codes. *W* will be used to denote a communication channel with binary input $x\in F_{2}$ and output from an alphabet y∈Y. In particular, we will focus on BEC(p), where the output is Y={0,1,?}, ? denoting an erasure and *p* being the erasure probability. For a more detailed reading of the subject, we recommend [35,36].

### 2.1. Monomial Codes

Monomial codes are a special class of structured codes. Informally, any code that admits a basis, in which each vector is the evaluation of a monomial, is called a monomial code. In general, monomial codes have a predefined length, i.e., $2^{m}.$ Many of the notations, definitions, properties, and results presented in this section are taken from [3].

In this article, binary vectors of length *m* will be denoted using bold small letters, e.g., $u=\left(u_{0},\cdots ,u_{m-1}\right)\in F_{2}^{m}$, with the convention that bits are ordered from left to right, $u_{0}$ being the least significant bit. We also define the bit-wise complement of $u\in {\left\{0,1\right\}}^{m}$ by $\bar{u}=1_{m}⊕u$ (as in [4]), where $1_{m}$ is the all-ones vector. The set $\left\{u\in F_{2}^{m}\right\}$ will be ordered in a natural manner, using the mapping

$$\left(u_{0},\cdots ,u_{m-1}\right)\to u=\sum_{i=0}^{m-1}u_{i}2^{i},$$

and the natural order on the integers. Notice that we compute the value *u* regardless of the fact that $u_{i}\in F_{2}.$ Notice that the relation between u and $\bar{u}$ induces $u+\bar{u}=2^{m}-1$.

We consider multivariate polynomials and monomials defined over the polynomial ring $R_{m}=F_{2}\left[x_{0},x_{1},\cdots ,x_{m-1}\right]/\left(x_{0}^{2}-x_{0},\cdots ,x_{m-1}^{2}-x_{m-1}\right).$ The usual operators will be employed, i.e., for $f,g\in R_{m}$, we denote by degf the degree of *f*, gcd(f,g) the greatest common divisor of *f* and *g*. f/g denotes the quotient of *f* and *g*.

**Notation** **1.** *Let m be a strictly positive integer. We denote*

- *monomials: $x^{u}=x_{0}^{u_{0}}\cdots x_{m-1}^{u_{m-1}},$ where $u\in F_{2}^{m}.$*
- *support of a monomial: $\mathrm{ind}\left(g\right)=\left\{l_{1}\;\cdots ,l_{s}\right\}$, where $g=x_{l_{1}}\cdots x_{l_{s}}$ and $0\leq l_{1}<l_{2}\cdots <l_{s}\leq m-1.$*
- *a subset of the support of a monomial: $g_{\left[0,s\right]}=\gcd\left(g,\prod_{i=0}^{s}x_{i}\right).$*
- *the set of monomials: $M_{m}\overset{\mathrm{def}}{=}\left\{x^{u}\;|\;u=\left(u_{0},\cdots ,u_{m-1}\right)\in F_{2}^{m}\right\}.$*

**Proposition** **1** ([37]). *Let $g\in R_{m}$ and order the elements in $F_{2}^{m}$ with respect to the decreasing index order. Define the evaluation function*

$$\begin{array}{ccc} R_{m} & \to & F_{2}^{2^{m}}\\ g & \mapsto & \mathrm{ev}\left(g\right)={\left(g(u)\right)}_{u\in F_{2}^{m}} \end{array}$$

*Then, ev is a bijection defining an isomorphism between the vector spaces $\left(R_{m},+,\cdot \right)$ and $(F_{2}^{n},+,\cdot ).$*

Now, we are ready to define the concept of monomial codes.

**Definition** **1** (Monomial code). *Let $I\subseteq M_{m}$ be a finite set of monomials in m variables. The linear code defined by I is the vector subspace $C\left(I\right)\subseteq F_{2}^{2^{m}}$ generated by {ev(f)|f∈I} that is called monomial code.*

**Proposition** **2** ([3]). *For all $I\subseteq M_{m}$, the dimension of the monomial code C(I) is equal to |I|.*

**Remark** **1.** *The $r^{th}$ order Reed–Muller code $\mathrm{RM}\left(r,m\right)\overset{def}{=}\left\{\mathrm{ev}\left(g\right)\;|\;g\in R_{m},\deg g\leq r\right\}$ is a monomial code with dimension k=∑i=0rmi.*

### 2.2. Polar Codes

In order to define polar codes, we have to introduce the concept of synthetic channels. Consider the channel transformation $W\to (W_{2}^{\left(0\right)},W_{2}^{\left(1\right)})$ defined in the following manner.

**Definition** **2** (Synthetic channels). *Let W be a BDMC with output alphabet Y and $x_{1},x_{2}\in F_{2}$ be the inputs and $y_{1},y_{2}\in Y$ be the outputs of two copies of W. Define two new channels*

$$\begin{array}{ccc} W^{\left(1\right)}\left(y_{1},y_{2}|x_{2}\right) & \overset{def}{=} & \frac{1}{2}\sum_{x_{1}\in F_{2}}W\left(y_{1}|x_{1}\right)W\left(y_{2}|x_{1}⊕x_{2}\right)\\ W^{\left(0\right)}\left(y_{1},y_{2},x_{2}|x_{1}\right) & \overset{def}{=} & \frac{1}{2}W\left(y_{1}|x_{1}\right)W\left(y_{2}|x_{1}⊕x_{2}\right). \end{array}$$

For any $u=\left(u_{0},\cdots ,u_{m-1}\right)\in {\left\{0,1\right\}}^{m}$, we define $W^{u}={\left({\left(W^{u_{m-1}}\right)}^{\cdots }\right)}^{u_{0}}$ as in [4]. Moreover, we extend the notation to monomials, by $W_{m}^{f}=W^{u}$ where $f=x^{u}\in M_{m}.$ We are using the index *m* in $W_{m}^{f}$ to precisely identify the number of variables on which *f* is expressed. For example, if u=(1,0,0,1,1), we have $W_{5}^{f}=W_{5}^{x_{0}x_{3}x_{4}}={\left(W_{1}^{x_{4}}\right)}_{4}^{x_{0}x_{3}}={\left({\left(W_{1}^{x_{4}}\right)}_{1}^{x_{3}}\right)}_{3}^{x_{0}}.$

**Definition** **3.** *Let W be a BDMC with output alphabet Y. Then, the Bhattacharyya parameter of the channel W is*

(2) $$B\left(W\right)=\sum_{y\in Y}\sqrt{W(y|0)W(y|1)}.$$

**Remark** **2.** *Let W be a BEC(p), then, we have that $B\left(W^{x_{0}}\right)=B\left(W^{\left(1\right)}\right)=2p-p^{2}$ and $B\left(W^{1}\right)=B\left(W^{\left(0\right)}\right)=p^{2}.$*

**Definition** **4.** *The polar code of length $n=2^{m}$ and dimension k devised for the channel W is the linear code obtained by selecting the set of k synthetic channels with the smallest $B\left(W^{u}\right)$ values among all $u\in {\left\{0,1\right\}}^{m}$.*

*Moreover, we define the relation*

(3) $$f\leq g\Leftrightarrow u\leq v\Leftrightarrow B\left(W^{u}\right)\left(p\right)\leq B\left(W^{v}\right)\left(p\right),\forall p\in \left[0,1\right].$$

The relation (3) is called universal, i.e., two monomials f,g satisfying f≤g are always comparable for any *p* and any m. This property can be used when constructing polar codes by storing a table with all such monomials. However, there might be several monomials bigger than *f* which are not comparable pairwise (see, for example, [4,14]). Indeed, one can easily verify that ≤ is a well-defined order relation (reflexive, anti-symmetric, and transitive), and thus, induces a poset on the set of monomials. In some particular cases, the order ≤ becomes total (all elements are ordered in a chain), e.g., when W=BEC and m≤4. However, in general, ≤ is a partial order, even in the case of W=BEC (starting from m=5), as pointed out in [3,11,30].

**Proposition** **3.** *Let m≥5 be an integer and W=BEC. Then, $\left\{M_{m},\leq \right\}$ is a poset.*

For simplification, when we refer to ordering the Bhattacharyya parameters, we will just write $B\left(W^{u}\right)\leq B\left(W^{v}\right).$

### 2.3. Weakly Decreasing and Decreasing Monomial Codes

**Definition** **5.** *Let f and g be two monomials in $M_{m}.$*

- *The ${⪯}_{w}$ order between f and g is defined as*
  $$f{⪯}_{w}g\;\mathrm{iff}\;f|g.$$
- *The ⪯ order between f and g is defined as*
  - *when deg(f)=deg(g)=s and $f=x_{i_{1}}\cdots x_{i_{s}}$, $g=x_{j_{1}}\cdots x_{j_{s}}$ we have*
    $$f⪯g\;\mathrm{iff}\;\forall \;1\leq \ell \leq s\;i_{\ell }\leq j_{\ell }.$$
  - *when deg(f)<deg(g) we have*
    $$f⪯g\;\mathrm{iff}\;\exists g^{*}\in M_{m}\;s.t.\;f⪯g^{*}{⪯}_{w}g.$$

The two order relations ${⪯}_{w}$ and ⪯ are well defined. ${⪯}_{w}$ was already used in the case of polar codes, but in a completely different context by Mori and Tanaka in [17]. In their case, the purpose was to tighten the bounds of the error block probability of a polar code designed for the BEC family.

Notice that ${⪯}_{w}$ is weaker than ⪯, meaning that $\forall f,g\in M_{m}f{⪯}_{w}g\Rightarrow f⪯g.$ The inverse is not always true: taking, for example, $f=x_{0}x_{2}$ and $g=x_{1}x_{2}$ it follows by definition that f⪯g but 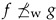. We also remark that 1 is the smallest element both for ⪯ and for ${⪯}_{w}$, and we have

$$1⪯x_{0}⪯x_{1}⪯\cdots ⪯x_{m-1}.$$

**Definition** **6.** *Let f and g be two monomials in $M_{m}$ such that f⪯g and $I\subset M_{m}.$*

- *We define the closed interval ${\left[f,g\right]}_{⪯}=\left\{h\in M_{m}\;|\;f⪯h⪯g\right\}.$*
- *$I\in M_{m}$ is called a decreasing set if and only if (f∈I and g⪯f) implies g∈I.*
- *Let $I\in M_{m}$ be a decreasing set. Then, C(I) is called a decreasing monomial code.*

Polar codes were recently related to network theory. In [30], the authors make a connection between the Bhattacharyya parameter of a synthetic channel and the reliability polynomial of a two-terminal network. Following the same path, we introduce in the next subsection all the required preliminaries in reliability and network theory.

### 2.4. Two-Terminal Networks

**Definition** **7.** *Let n be a strictly positive integer. We say that N is a two-terminal network (2TN) of size n if N is a network made of n identical devices, that has two distinct terminals: an input S, and an output T.*

To any network, N made of *n* devices we associate two parameters: width (*w*) and length (*l*), where *w* is the cardinal of a “minimal cut” separating *S* from *T*, and *l* is the cardinal of a “minimal path” from *S* to *T*, that satisfy

(4) n≥wl

(see Theorem 3 in [38]). The number of devices *n* is known in the literature as the size of the network. When n=wl, we say that N is a minimal 2TN [38].

**Figure 3:**
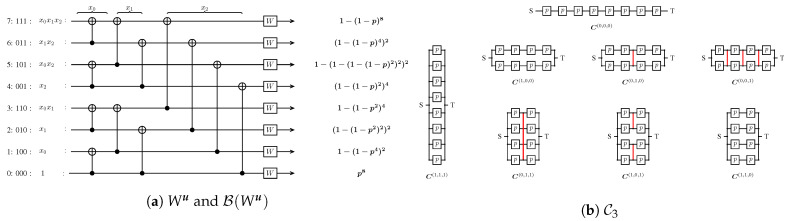
Combined circuit as defined by Arikan [1], the Bhattacharyya parameter of the corresponding synthetic channels and the compositions in $C_{3}$.

The composition of $N_{1}$ and $N_{2}$ can be defined as in [38]. The resulting network is obtained by replacing each device in $N_{1}$ by a copy of $N_{2}$. We will denote a composition by C, the simplest possible being two devices in series $C^{\left(0\right)}$, and two devices in parallel $C^{\left(1\right)}$. The composition of $C^{\left(0\right)}$ with $C^{\left(1\right)}$ is $C^{u}=C^{\left(0\right)}•C^{\left(1\right)}$, where u=(0,1). The set of all $2^{m}$-size compositions will be denoted by $C_{2^{m}}$, and the set of all compositions of width $2^{i}$ and length $2^{m-i}$ by $C_{2^{i},2^{m-i}}$ (see Figure 3b).

**Proposition** **4** ([39]). *Let m>0 and $C^{u}\in C_{2^{m}}.$ Then, $C^{u}$ is a minimal 2TN of size $2^{m},$ length $l=2^{m-|u|}$ and width $w=2^{\left|u\right|}.$ We also have $C_{2^{m}}={⋃}_{i=0}^{m}C_{2^{i},2^{m-i}}.$*

**Theorem** **3** ([11]). *There is a natural bijection between $C_{2^{m}}$ and the set of all $W^{u}$, for any fixed positive integer m.*

In Figure 3, we illustrate the bijection between the two aforementioned sets. More significant is the equality between the reliability polynomial of a composition $C^{u}$ and the Bhattacharyya parameter of $W^{u}$, a fact that is visible from Figure 3b and proven in the next paragraph.

#### Reliability Polynomial

The reliability of N is defined as the probability that *S* and *T* are connected (also known as s,t-connectivity) [40]. One of the most common hypotheses considered in network theory is that devices are uniformly and identically supposed to close with a probability p∈[0,1]. Hence, the reliability of N, denoted by Rel(N;p), can be expressed as a polynomial

(5) $$\begin{array}{cc} \mathrm{Rel}(N;p) & =\sum_{i=0}^{n}N_{i}\left(N\right)\;p^{i}{\left(1-p\right)}^{n-i}. \end{array}$$

The coefficients $N_{i}\left(N\right)$ represent the number of paths from *S* to *T* of length *i*. Several properties regarding the coefficients $N_{i}\left(N\right)$, as well as complementarity relations between a 2TN N and its dual $N^{⊥}$, are detailed in [32,41] in the case of hammock networks.

### 2.5. Bhattacharyya Parameters and Reliability Polynomials

**Theorem** **4** ([11]). *Let m>0, $u\in {\left\{0,1\right\}}^{m}$, and W=BEC(p)*

(6) $$B\left(W^{u}\right)\left(p\right)=\mathrm{Rel}\left(C^{u};p\right)$$

*where $\mathrm{Rel}\left(C^{\left(0\right)};p\right)=p^{2}$ and $\mathrm{Rel}\left(C^{\left(1\right)};p\right)=1-{\left(1-p\right)}^{2}$.*

**Proposition** **5** ([25]). *Let m>0 and $u\in {\left\{0,1\right\}}^{m}.$ Then*

(7) $$B\left(W^{\bar{u}}\right)\left(p\right)=1-B\left(W^{u}\right)\left(1-p\right).$$

This condition expresses the duality of the two corresponding networks, namely $C^{\bar{u}}$, the dual of $C^{u}$ (see [11,32]). Notice that by (7), one has to analyze only u with |u|≤m/2.

## 3. Polar Codes Are Strongly Decreasing Monomial Code Over the BEC

### 3.1. Definitions and Results

**Definition** **8.** *The ${⪯}_{d}$ order between f and g is defined as*

- *when deg(f)=deg(g)=s and $f=x_{i_{1}}\cdots x_{i_{s}}$, $g=x_{j_{1}}\cdots x_{j_{s}}$ we have*
  $$f{⪯}_{d}g\;\mathrm{iff}\;\forall \ell \in \left\{1,\cdots ,s\right\}\mathrm{we}\;\mathrm{have}\sum_{k=0}^{\ell }i_{s-k}\leq \sum_{k=0}^{\ell }j_{s-k}.$$
- *when deg(f)<deg(g) we have*
  $$f{⪯}_{d}g\;\mathrm{iff}\;\exists g^{*}\in M_{m}\;s.t.\;f{⪯}_{d}g^{*}{⪯}_{w}g.$$

**Definition** **9.** *Let f and g be two monomials in $M_{m}$, such that f⪯g and $I\subset M_{m}.$*

- *We define the closed interval ${\left[f,g\right]}_{{⪯}_{d}}=\left\{h\in M_{m}\;|\;f{⪯}_{d}h{⪯}_{d}g\right\}.$*
- *$I\in M_{m}$ is called a strongly decreasing set if, and only if, (f∈I and $g{⪯}_{d}f$) implies g∈I.*
- *Let $I\in M_{m}$ be a strongly decreasing set. Then, C(I) is called strongly decreasing monomial code.*

**Lemma** **1.** *The order ${⪯}_{d}$ is a well-defined order relation and $\left\{M_{m},{⪯}_{d}\right\}$ forms a Poset.*

The proof of this lemma comes directly from the definition of ${⪯}_{d}$.

**Remark** **3.** *Notice that $x_{i_{1}}\cdots x_{i_{s}}⪯x_{j_{1}}\cdots x_{j_{s}}$ implies that $x_{i_{1}}\cdots x_{i_{s}}{⪯}_{d}x_{j_{1}}\cdots x_{j_{s}}.$ The converse is no longer true, take for example, the monomials $x_{0}x_{3}$ and $x_{1}x_{2}.$*

**Proposition** **6.** *Let f and g be two monomials with the same degree and $x_{h}$ be such that $x_{h}$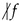 and $x_{h}$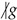. Then, we have*

(8) $$f{⪯}_{d}g\;\mathrm{iff}\;x_{h}f{⪯}_{d}x_{h}g.$$

The proof of Proposition 6 can be found in Appendix A. In particular, notice that if f,g are co-prime, i.e., gcd(f,g)≠1, then

(9) $$f{⪯}_{d}g\;\mathrm{iff}\;f/\gcd\left(f,g\right){⪯}_{d}g/\gcd\left(f,g\right).$$

Remark that when the condition on variable $x_{h}$ is not satisfied, the result does not hold, e.g., $x_{2}x_{3}{⪯}_{d}x_{1}x_{4}$, but $x_{1}x_{2}x_{3}$ and $x_{1}x_{4}$ are not comparable with respect to ${⪯}_{d}.$ Before we get to our main theorem of this section, the following lemma is required.

**Lemma** **2.** *Let $f,g\in M_{m},\deg f=\deg g=2$, such that $f{⪯}_{d}g.$ Then, $B\left(W_{m}^{f}\right)\leq B\left(W_{m}^{g}\right).$*

**Corollary** **1.** *Let $f=x_{i_{1}}x_{i_{2}}$ and $g=x_{j_{1}}xj_{2}$ s.t. $f{⪯}_{d}g.$ Then, for any monomial $h=x_{l_{1}}\cdots x_{l_{t}}$ satisfying $i_{1}<l_{1}<\cdots <l_{t}<i_{2}$, we have $fh{⪯}_{d}gh$ and $B\left(W_{m}^{fh}\right)\leq B\left(W_{m}^{gh}\right).$*

**Theorem** **5.** *Polar codes over the binary erasure channel are strongly decreasing monomial codes.*

The proof of Theorem 5 is given in Appendix A.

**Theorem** **6.** *Reed–Muller codes are strongly decreasing monomial codes, i.e.,*

(10) $$\mathrm{RM}\left(i,m\right)=C({\left[1,x_{m-i}\cdots x_{m-1}\right]}_{{⪯}_{d}}).$$

**Proof.**  The proof follows from ${\left[1,x_{m-i}\cdots x_{m-1}\right]}_{{⪯}_{d}}={\left[1,x_{m-i}\cdots x_{m-1}\right]}_{⪯}$ and $\mathrm{RM}\left(i,m\right)=C({\left[1,x_{m-i}\cdots x_{m-1}\right]}_{⪯})$ (see Proposition 3.3.12 in [3]). □

### 3.2. Perspectives of Application of ${⪯}_{d}$ in the Construction of Polar Codes

State-of-the-art algorithms for constructing polar codes [14] are using the structure induced by the existing partial order relations on the set of monomials. As explained in [14], the complexity of the algorithm for construction of polar codes is dominated by the cardinality of the largest set of non comparable monomials with respect to ⪯. Hence, a finer order relation than ⪯ could potentially decrease the complexity of such an algorithm. As ${⪯}_{d}$ is thinner than ⪯, we will seek, through examples, how many non-comparable monomials with respect to ⪯ are comparable with respect to ${⪯}_{d}.$ Typically, our procedure can be used for a more efficient enumeration of sets of non-comparable elements in the new poset $\{M_{m},{⪯}_{d}\}.$ The longest antichain in the poset gives a direct intuition on how efficient the construction algorithm can be. Indeed, when estimating the reliability of the synthetic channels in order to construct a polar code, one need to estimate the reliability of the non comparable elements. Hence, having a finner poset, where the maximum length antichain becomes smaller, induces a more efficient construction algorithm. In order to make things clear, we will explain, in view of two distinct perspectives (reducing the number of non comparable elements and reducing the number of codes of fixed dimension), how ${⪯}_{d}$ induces more efficient construction rules for the polar code.

#### 3.2.1. Reducing the Number of Non-Comparable Monomials

**Figure 4:**
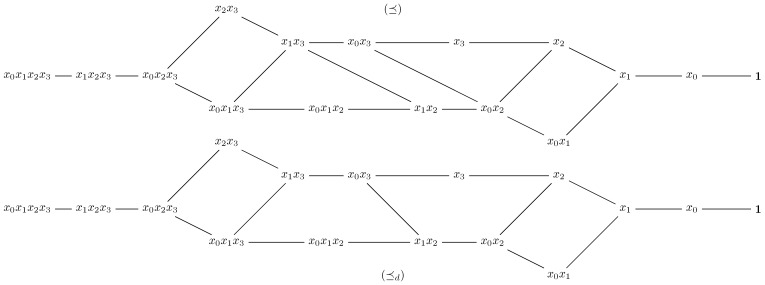
The two-order relations ⪯ and ${⪯}_{d}$ for m=4.

For $\left\{M_{m},⪯\right\}$, the middle of the poset is also a maximum length antichain. Let us explain the concept of middle of $\{M_{m},⪯\}.$ A monomial *g* is situated in the middle of the poset if any chain from *g* to 1 (the infimum of the poset) has length equal to any chain from *g* to $x_{0}\cdots x_{m-1}$ (the supremum of the poset). Clearly, two distinct monomial f,g that are in the middle of the poset are non-comparable. Moreover, notice that not every poset admits a middle, with respect to our definition. For example, $\left\{M_{m},⪯\right\}$ has a middle, since it is a graded poset (see [11,14,31] for more details). However, $\left\{M_{m},{⪯}_{d}\right\}$ is not graded and it does not admit a middle. When m=4 (see Figure 4), the middle of $\left\{M_{m},⪯\right\}$ is the set $\{x_{1}x_{2},x_{0}x_{3}\}.$ As ${⪯}_{d}$ is thinner than ⪯, it could be possible to reduce the number of non-comparable elements from the middle of $\{M_{m},⪯\}.$ Indeed, this fact can be validated through simulations, as we point out in Table 1.

##### Simulations

We have implemented an algorithm for generating all elements in the middle of $\{M_{m},⪯\}.$ For each value of *m* in {6..8}, the elements in the set are displayed in Table 1. We choose to display the Shift(ind(g)) instead of *g*, where for $g=x_{i_{1}}\cdots x_{i_{l}}$, $\mathrm{Shift}\left(\mathrm{ind}\left(g\right)\right)=\left(i_{1}+1,\cdots ,i_{l}+1\right).$ The main reason for this convention is that the elements in the middle of $\left\{M_{m},⪯\right\}$ are the answers of a well-known problem in computer science, i.e., perfect subset sum problem. Indeed, if we carefully check the elements for each value of *m*, we discover that ∑j∈Shift(ind(g))j=⌊m+12/2⌋ for any *g* in the middle of the poset $\{M_{m},⪯\}.$

As one can notice from Table 1, the number of non comparable elements from the middle of $\left\{M_{m},⪯\right\}$ decreases rapidly when the order relation ${⪯}_{d}$ is used. For example, when m=8, we have decreased this number from 14 non comparable monomials, with respect to ⪯, to 3 distinct non comparable chains, with respect to ${⪯}_{d}.$

#### 3.2.2. Reducing the Number of Codes

Another pertaining aspect when we deal with decreasing and strongly decreasing monomial codes is the estimation of codes for a fixed length $2^{m}$ and dimension $k\in \{1..2^{m}\}.$ It seems quite natural, in view of the relation between ⪯ and ${⪯}_{d}$, to state that there are fewer strongly decreasing monomial codes of fixed length and dimension than decreasing monomial codes. Formally, we have

**Lemma** **3.** *Let m be a strictly positive integer and $0\leq k\leq 2^{m}.$ Then the number of decreasing monomial code C(I) with $I\in M_{m}$ and |I|=k greater than or equal to the number of strongly decreasing monomial codes C(J) with $J\in M_{m}$ and |J|=k.*

The proof of this lemma is obvious and comes directly from the fact that any strongly decreasing monomial set *I* is necessarily a decreasing monomial set. However, the inverse is not always true.

##### Simulations

We have written an algorithm that computes the number of decreasing monomial codes for a fixed length and dimension. Our algorithm works recursively, by adding new monomials from {Mon,⪯} to the previous sets of monomials of cardinality k−1, in such a manner that the cardinality of the new sets does not exceed k. The algorithm outputs all possible decreasing monomials sets of cardinality *k* and then it checks which of them are also strongly decreasing monomial sets.

For small values of *k*, i.e., k=O(1) when n→∞, there are almost no differences between strongly decreasing and decreasing sets. However, for bigger values of *k*, we observe a significant reduction of the number of strongly decreasing monomial codes compared to decreasing monomial codes. In Table 2, we illustrate on a small example m=5 and k∈{9,10,11,12} the difference between the two sets. We choose to represent each monomial set *I* by ind(I)={ind(g),g∈I}.

## 4. Average Reliability of the Synthetic Channels

The geometric approach of the properties of a function by means of its subgraph and/or epigraph generated useful mathematical tools from the very beginning of the theory of functions. Measure, intersection, support and shape properties lead to applications in various domains: optimization, shape description and recognition, etc. Here, we propose a geometric approach in the field of polar coding. Recently, the concept of average reliability was introduced and analyzed in the context of all terminal reliability [42]. In view of Theorem 4, the Bhattacharyya parameter of a synthetic channel can be mapped into the reliability polynomial of a minimal two-terminal network. As a consequence, almost all constructive and efficient methods from network reliability can be applied to polar codes over BEC, by means of the Bhattacharyya parameter.

As the set of the synthetic channels cannot be totally ordered [4,25], we propose a different method to define the optimality of a synthetic channel. For that, we will check how reliable a channel is on average, i.e., we define

**Definition** **10.** *Let m be a strictly positive integer and $u\in {\left\{0,1\right\}}^{m}$. The average reliability of $W^{u}$ is*

$$\mathrm{Avr}\left(W^{u}\right)=\int_{0}^{1}B\left(W^{u}\right)\left(p\right)dp.$$

*Moreover, we define the relation $\leq_{\mathrm{Avr}}$*

$$u\leq_{\mathrm{Avr}}v\Leftrightarrow \mathrm{Avr}\left(W^{u}\right)\leq \mathrm{Avr}\left(W^{v}\right)$$

This notion of optimality has a meaning in the following context. Imagine that the communication channel is a BEC with variable erasure probability, coming from different physical reasons. This means that either we choose a different polar code in function of the variations of *p* and in this case we obtain the best performance for each instance, or we choose a polar code and hope that on average it performs in an optimal way. The former strategy comes with the cost of computing for each value of *p* the corresponding polar code; as for the latter, the cost is minimal, since we construct only one polar code.

### 4.1. Properties

**Lemma** **4.** *The relation $\leq_{\mathrm{Avr}}$ is reflexive and transitive. In other words, $\leq_{\mathrm{Avr}}$ is a preorder relation.*

Our simulations have shown that up to m=13, $\leq_{\mathrm{Avr}}$ is also antisymmetric. However, this property might not be true in general. Indeed, one can easily find two distinct polynomials with integer coefficients defined over [0,1] with values in [0,1], such that their integrals are equal.

**Conjecture** **1.** *The relation $\leq_{\mathrm{Avr}}$ is a preorder relation on the set of all synthetic channels.*

All the same, we can overcome this by applying the following procedure.

**Remark** **4.** *Let $u\equiv_{\mathrm{Avr}}v$ if, and only if, $\mathrm{Avr}\left(B\left(W^{u}\right)\right)=\mathrm{Avr}\left(B\left(W^{v}\right)\right).$ Let us extend the relation $\leq_{\mathrm{Avr}}$ to the factor set $M_{m}/\equiv_{\mathrm{Avr}}$ naturally, using the relation between class representatives. Then, $\leq_{\mathrm{Avr}}$ is a total order relation over $M_{m}/\equiv_{\mathrm{Avr}}.$ Indeed, one can easily check that $\leq_{\mathrm{Avr}}$ is antisymmetric over $M_{m}/\equiv_{\mathrm{Avr}}.$*

**Lemma** **5.** *Let m be a strictly positive integer, $n=2^{m}$, and $u\in {\left\{0,1\right\}}^{m}.$ Then,*

(11) AvrWu=1n+1∑i=2m−|u|nNi(Cu)ni.

(12) $$\mathrm{Avr}\left(W^{u}\right)+\mathrm{Avr}\left(W^{\bar{u}}\right)=1.$$

**Proposition** **7.** *Let m be a strictly positive integer and u,v be two binary vectors of length m, such that u≤v. Then,*

(13) $$u\leq v\Rightarrow \mathrm{Avr}\left(W^{u}\right)\leq \mathrm{Avr}\left(W^{v}\right).$$

In Table 3, we compute the $\mathrm{Avr}\left(W^{u}\right)$ of all the binary vectors $u\in {\left\{0,1\right\}}^{m}$ for m∈{2,3,4}. Notice that in this case, Proposition 7 applies, since we know that up to m=4, the synthetic channels can be totally ordered over the BEC [3,25]. Starting from m=5, this property is no longer true. When u and v are no longer comparable, i.e., there is $p_{0}\in \left(0,1\right)$ such that $B\left(W^{u}\right)\left(p_{0}\right)=B\left(W^{v}\right)\left(p_{0}\right)$, we can still decide whether on average u is optimal compared with v. The set of non-comparable pairs (u,v) for m=5 is {(3,16),(12,17),(7,20),(7,24),(11,24),(14,19),(15,28)}. Notice that half of the pairs are coming from duality, i.e., if (u,v) are not comparable, then $\left(\bar{u},\bar{v}\right)$ are also non-comparable. However, these are ordered with respect to average reliability. The average reliability for the first 4 non-comparable pairs are (0.221,0.216),(0.396,0.383),(0.4712,0.4710),(0.4712,0.5288). Hence, for m=5 the ordering with respect to the average reliability is 0,1,2,4,8,16,3,5,6,9, 10,17,12,18,20,7,24, and the rest can be completed by symmetry.

**Example** **1.** *The ordering induced by the average reliability.*

- m=5
  $$0,\underset{\mathrm{RM}(1,5)}{\underbrace{1,2,4,8,16}},\overset{\mathrm{RM}(2,5)}{\overbrace{3,5,6,9,10,17,12,18,20}},\underset{\mathrm{RM}(3,5)}{\underbrace{7}},\overset{\mathrm{RM}(2,5)}{\overbrace{24}}$$
- m=6
  $$0,\underset{\mathrm{RM}(1,6)}{\underbrace{1,2,4,8,16}},\overset{\mathrm{RM}(2,6)}{\overbrace{3,5}},\underset{\mathrm{RM}(1,6)}{\underbrace{32}},\overset{\mathrm{RM}(2,6)}{\overbrace{6,9,10,17,12,18,33,20}},\underset{\mathrm{RM}(3,6)}{\underbrace{7}},\overset{\mathrm{RM}(2,6)}{\overbrace{34,24}},\underset{\mathrm{RM}(3,6)}{\underbrace{11}},\overset{\mathrm{RM}(2,6)}{\overbrace{36}},\underset{\mathrm{RM}(3,6)}{\underbrace{13,19,14}},\overset{\mathrm{RM}(2,6)}{\overbrace{40}},$$
  $$\underset{\mathrm{RM}(3,6)}{\underbrace{21}},\overset{\mathrm{RM}(2,6)}{\overbrace{48}},\underset{\mathrm{RM}(3,6)}{\underbrace{22,35,25,37,26,38,28,41}}$$

**Figure 5:**
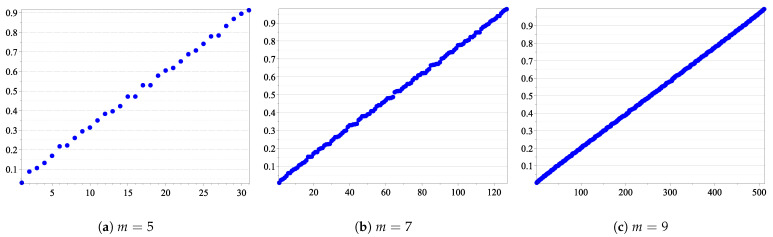
Sorted $\mathrm{Avr}\left(B\left(W^{u}\right)\right)$ for all ***u*** ∈ {0, 1}^*m*^.

Our simulations have shown that, considering the relation $\leq_{\mathrm{Avr}}$ in the set of the synthetic channels, in each sub-interval (i/10,(i+1)/10), for 0≤i≤9, we have a rough proportion of $2^{m}/10$ binary vectors u. So, roughly speaking, a uniform distribution could be used to approximate the number of u inside each sub-interval (illustrated in Figure 5), with respect to Avr (see Table 4 for 5≤m≤11.)

### 4.2. Relation to β-Expansion

β-expansion [15] is a well-known method for an efficient construction of polar codes. Hence, it is with no surprise that our results on average reliability determine possibly more refined choices of the variable β. Let us begin by defining the method.

(14) $$\beta \left(u\right)=\sum_{i=0}^{m-1}u_{i}\beta^{i}$$

In [15], the authors proved that for any β∈(1,∞), the order induced by β on the sequence of synthetic channels respects the order relation ⪯. In particular, this means that if u⪯v then β(u)≤β(v) and this for any value of β>1. Some values of β are of high interest, in particular $\beta =2^{1/4}$, when *W* is designed for additive white Gaussian noise (AWGN). In the case of AWGN, the authors in [15] proposed a procedure in which an interval for β is determined, an interval that converges to a value close to $2^{1/4}.$ Notice that in [15], the order induced by β is not valid for any signal to noise ratio value, but it tries to cover as much as possible the interval [0,1]. A natural question that one could raise is whether there is a β-expansion for the average reliability, i.e., is there a real value β such that β and Avr are identical over the set of binary vectors of length m. There is a significant difference between the two relations. In our case, not only that *W* is a BEC, but also the preorder induced by the average reliability is total over $M_{m}/\equiv_{\mathrm{Avr}}$ and holds for the entire interval [0,1].

**Remark** **5.** *By computer simulations, one can easily prove that for m≤5, there is β∈(1,∞), such that the order induced by beta and the preorder induced by the average reliability coincide. It can be done by simply tacking β=1.22.*

**Conjecture** **2.** *For m>6, we did not find a value of β for which the two aforementioned relations are equal. Moreover, for β∼1.22, the number of elements with similar mutual relations with respect to the two relations is minimized (see Table 5).*

### 4.3. Threshold Points of the Binary Erasure Polarization Sub-Channels

The fact that when *m* goes to infinity the Bhattacharyya polynomial has a sharp transition from zero to one when *m* goes to infinity has already been proven ([34]). More exactly, for any u, there exists a point $p_{0}\left(u\right)\in \left(0,1\right)$ for which in its vicinity $B\left(W^{u}\right)$ passes from very small values (close to zero) to very high values (close to one). Formally speaking, we have

**Lemma** **6** ([34]).

(15) $$\lim_{m\to \infty }B\left(W^{u}\right)=\left\{\begin{array}{cc} 0 & p\in [0,p_{0}\left(u\right))\\ 1 & p\in (p_{0}\left(u\right),1] \end{array}\right.$$

However, finding the point $p_{0}\left(u\right)$ where this transition holds is not trivial (see [15,29]). Here, we will use the average reliability to determine this point for some specific channels.

**Lemma** **7.** (16) $$\lim_{m\to \infty }\mathrm{Avr}\left(W^{u}\right)=1-p_{0}\left(u\right).$$

A particular interesting channel analyzed in [25,29] is the synthetic channel $W^{\left(1^{i}0^{m-i}\right)}.$ More exactly, the authors analyze the sharp transition of $W^{\left(1^{i}0^{m-i}\right)}$ from 0 to 1 when *m* tends to infinity, in function of the limit i/m−i. Here, we will give an exact formula for the average reliability of $W^{{(}^{\prime }1^{i}0^{m-i})}$. This result combined with Lemma 7 will allow us to obtain a finer approximation of $p_{0}\left(u\right).$ To achieve our goal, we will look at the corresponding 2TN, namely at $C^{\left(1^{i}0^{m-i}\right)}$. For simplification, we use $l=2^{m-i},w=2^{i}$ and $n=2^{m}.$ Notice that

(17) $$\mathrm{Rel}\left(C^{\left(1^{i}0^{m-i}\right)};p\right)=1-{\left(1-p^{l}\right)}^{w}.$$

**Theorem** **7.** (18) RelC(1i0m−i);p=∑i=ln∑j=1⌊il⌋(−1)j+1wjn−jln−ipi(1−p)n−i.

**Proof.**  In order to prove our result, we need to demonstrate that ∀l≤i≤n

(19) Ni(C(1i0m−i))=∑j=1⌊il⌋(−1)j+1wjn−jln−i

The proof is based on an inclusion-exclusion argument. Denote by $P_{i}$ the set of paths of length *i* from *S* to *T* for the $C^{\left(1^{i}0^{m-i}\right)}$. This leads to $\left|P_{i}\right|=N_{i}\left(C^{\left(1^{i}0^{m-i}\right)}\right).$

Any path of length *i* with l≤i is composed of at least one path of length *l*, hence we have *w* choices for fixing a path of length *l* and n−li choices for the remaining positions. However, in the n−li choices, we might count other *l* length paths. Hence, we need to subtract the over-counting, which is all the combinations of two length *l* paths, i.e., w2, times the number of choices for the remaining positions, i.e., n−2li−2l. Now, we need to add all the paths that are composed of at least 3 *l* paths which equals w3n−3li−3l, and so on till we reached the last level, i.e., w⌊il⌋n−l⌊il⌋i−l⌊il⌋. □

**Theorem** **8.** (20) AvrW(1i0m−i)=1−12i+2i−m2i

**Proof.**  AvrWu=1n+1∑i=lnNi(Cu)ni=1n+1∑i=ln∑j=1⌊il⌋(−1)j+1wjn−jln−ini=1n+1∑j=1w∑i=jln(−1)j+1wjn−jln−ini=1n+1∑j=1w∑i=jln(−1)j+1wjijlnjl=1n+1∑j=1w(−1)j+1wjnjl∑i=jlnijl=1n+1∑j=1w(−1)j+1wjn+1jl+1njl=∑j=1w(−1)j+1wj1jl+1=1−∑j=0w(−1)jwj1jl+1=1−1n+1lw

□

Basically, we have

**Corollary** **2.** (21) AvrW(0i1m−i)=12i+2i−m2i

Based on Theorem 8, we can establish new classes of asymptotically “good” channels. For that, we will need the following result.

**Lemma** **8.** (22)limn→∞nlog2(n)(log2(log2(n)))+1log2(n)(log2(log2(n)))nlog2(n)(log2(log2(n)))=1.(23)limn→∞nlog2(log2(n))+1log2(log2(n))nlog2(log2(n))=∞.(24)limn→∞nlog2(n)+1log2(n)nlog2(n)=2.

Theorem 8, Lemma 8 and Lemma 7 imply the following result.

**Corollary** **3.** *Let m be a strictly positive integer and $u=\left(1^{i}0^{m-i}\right)$ Then,*

- *for any $i\leq m-\log_{2}\left(m\right)-\log_{2}\left(\log_{2}\left(m\right)\right)$ we have $p_{0}\left(u\right)\to 1$ and $p_{0}\left(\bar{u}\right)\to 0.$*
- *for any $i\geq m-\log_{2}\left(\log_{2}\left(m\right)\right)$ we have $p_{0}\left(u\right)\to 0$ and $p_{0}\left(\bar{u}\right)\to 1.$*

Another direct consequence of our results is that for any $i\leq m-\log_{2}\left(m\right)-\log_{2}\left(\log_{2}\left(m\right)\right)$, the monomial $f=x_{0}\cdots x_{i-1}$ is highly reliable on average. Hence, all the monomials $g{⪯}_{d}f$ are also highly reliable in average, as their average reliability tends to zero when *m* goes to infinity. Moreover, *f* becomes unreliable on average for $i\geq m-\log_{2}\left(\log_{2}\left(m\right)\right).$ The values $m-\log_{2}\left(m\right)-\log_{2}\left(\log_{2}\left(m\right)\right)<i<m-\log_{2}\left(\log_{2}\left(m\right)\right)$ are to be considered in more detail.

**Corollary** **4.** *Let m be a strictly positive integer and $i\leq \log_{2}\left(\log_{2}\left(m\right)\right).$ Then, for any $f\in M_{m}$ with $f{⪯}_{d}x_{m-i+1}\cdots x_{m}$, we have that $\mathrm{Avr}(W^{f})\to 0$ when m→∞. In other words, any synthetic channel in the RM(i,m) is asymptotically “good” on average.*

## 5. Conclusions and Perspectives

A complete characterization of the Bhattacharyya parameter of synthetic channels of a monomial code is an open problem that has attracted a lot of attention in the last decade. Even for the particular case of binary erasure channel, the question remains unanswered. However, the implications of such a result are of high importance in coding theory, especially in polar coding. In this article, we make a step forward by proposing an order relation ${⪯}_{d}$ that decreases the gap between state-of-the-art and the ultimate partial order relation for the Bhattacharyya parameter of synthetic channels. The advantage of this approach is that our algebraic description is rather easy to implement and analyze, compared to other order relations such as [25]. Simulations show that ${⪯}_{d}$ is a valid order relation on binary symmetric channel, and a deeper inspection of [25], and our work could potentially determine an algebraic description that fits the latest results.

The order relation proposed here could be employed as a new construction rule for polar codes. As ${⪯}_{d}$ is thinner than ⪯, it enables two reductions:

- the number of non-comparable monomials in the middle of $\left\{M_{m},⪯\right\}$ is significantly reduced by means of ${⪯}_{d}$,
- the number of strongly decreasing monomial codes is less than the number of decreasing monomial codes for fixed length and dimension.

Hence, these two properties open the perspectives of a more efficient construction algorithm for strongly decreasing monomial codes, and hence for polar codes.

As the relations on the Bhattacharyya parameter are all partial orders, we have proposed an alternative solution for ordering the synthetic channels. For that, we have used the concept of average reliability, borrowed from network theory. Instead of the local evaluation of the Bhattacharyya parameter, we propose a global one, by evaluating the integral, i.e., by measuring its global average behavior. Hence, we rank the synthetic channels using a preorder relation $\leq_{\mathrm{Avr}}$, given by the value of the integral. Our result is not constructive, in the sense that it does not fully characterize the channels that belong to a specific interval. An answer to this question might provide an extremely efficient method for constructing polar codes and give much more insight into the synthetic channels $W^{u}.$

## Figures and Tables

**Table 1:** Non comparable elements in the middle of $\left\{M_{m},⪯\right\}$.

m=6			
⪯	(4,3,2,1),(5,3,2),(5,4,1),(6,3,1),(6,4)		
${⪯}_{d}$	(4,3,2,1)		
	$\left(5,3,2\right){⪯}_{d}\left(5,4,1\right){⪯}_{d}\left(6,3,1\right)$		
	(6,4)		
m=7			
⪯	(5,4,3,2),(6,4,3,1),(6,5,2,1),(6,5,3),(7,4,2,1),(7,4,3),(7,5,2),(7,6,1)		
${⪯}_{d}$	$\left(5,4,3,2\right){⪯}_{d}\left(6,4,3,1\right){⪯}_{d}\left(6,5,2,1\right){⪯}_{d}\left(7,4,2,1\right)$		
	$\left(6,5,3\right){⪯}_{d}\left(7,4,3\right){⪯}_{d}\left(7,5,2\right){⪯}_{d}\left(7,6,1\right)$		
m=8			
⪯	(8,4,3,2,1),(7,5,3,2,1),(6,5,4,2,1),(8,7,3),(8,6,4),(7,6,5)		
	(6,5,4,3),(7,5,4,2),(7,6,3,2),(7,6,4,1),(8,5,3,2),(8,5,4,1),(8,6,3,1),(8,7,2,1)		
${⪯}_{d}$	$\left(6,5,4,2,1\right){⪯}_{d}\left(7,5,3,2,1\right){⪯}_{d}\left(8,4,3,2,1\right)$		
	$\left(7,6,5\right){⪯}_{d}\left(8,6,4\right){⪯}_{d}\left(8,7,3\right)$			
	$\left(6,5,4,3\right){⪯}_{d}\left(7,5,4,2\right){⪯}_{d}\left(7,6,3,2\right){⪯}_{d}$	(7,6,4,1)	${⪯}_{d}\left(8,5,4,1\right){⪯}_{d}\left(8,6,3,1\right){⪯}_{d}\left(8,7,2,1\right)$
		(8,5,3,2)	

**Table 2:** Decreasing and strongly decreasing monomial sets for m=5.

*k*	ind(*I*) for ⪯	ind(*J*) for ${⪯}_{d}$
9	{0,1,2,3,4,01,02,03,04}	
	{0,1,2,3,4,01,02,03,012}	
	{0,1,2,3,4,01,02,03,12}	{0,1,2,3,4,01,02,03,12}
	{0,1,2,3,01,02,03,12.012}	{0,1,2,3,01,02,03,12,012}
	{0,1,2,3,01,02,03,12,13}	{0,1,2,3,01,02,03,12,13}
10	{0,1,2,3,4,01,02,03,04,12}	
	{0,1,2,3,4,01,02,03,12,012}	{0,1,2,3,4,01,02,03,12,012}
	{0,1,2,3,4,01,02,03,12,13}	{0,1,2,3,4,01,02,03,12,13}
	{0,1,2,3,01,02,03,12,13,012}	{0,1,2,3,01,02,03,12,13,012}
	{0,1,2,3,01,02,03,12,13,23}	
11	{0,1,2,3,4,01,02,03,04,12,012}	
	{0,1,2,3,4,01,02,03,04,12,13}	{0,1,2,3,4,01,02,03,04,12,13}
	{0,1,2,3,4,01,02,03,12,13,012}	{0,1,2,3,4,01,02,03,12,13,012}
	{0,1,2,3,4,01,02,03,12,13,23}	
	{0,1,2,3,01,02,03,12,13,23,012}	
	{0,1,2,3,01,02,03,12,13,012,013}	{0,1,2,3,01,02,03,12,13,012,013}
12	{0,1,2,3,4,01,02,03,04,12,13,012}	{0,1,2,3,4,01,02,03,04,12,13,012}
	{0,1,2,3,4,01,02,03,04,12,13,23}	{0,1,2,3,4,01,02,03,04,12,13,23}
	{0,1,2,3,4,01,02,03,04,12,13,14}	
	{0,1,2,3,4,01,02,03,12,13,012,013}	{0,1,2,3,4,01,02,03,12,13,012,013}
	{0,1,2,3,4,01,02,03,12,13,23,012}	
	{0,1,2,3,01,02,03,12,13,23,012,013}	

**Table 3:** Average reliability of the synthetic channels.

m=2															
0	1	2	3												
0.20	0.47	0.53	0.80												
m=3															
0	1	2	4	3	5	6	7								
0.11	0.29	0.34	0.41	0.59	0.66	0.71	0.89								
m=4															
0	1	2	4	8	3	5	6	9	10	12	7	11	13	14	15
0.06	0.16	0.20	0.24	0.30	0.38	0.44	0.48	0.52	0.56	0.62	0.70	0.76	0.80	0.84	0.94

**Table 4:** Number of $u\in {\left\{0,1\right\}}^{m}$ that satisfy $\mathrm{Avr}\left(B\left(W^{u}\right)\right)\in \left(i/10,\left(i+1\right)/10\right]$, for 0≤i˂5, $\epsilon_{m}=2^{m-4}/10.$

*m*	(0,0.1]	(0.1,0.2]	(0.2,0.3]	(0.3,0.4]	(0.4,0.5]	[$\lfloor 2^{m}$/10-$\epsilon_{m}\rfloor ,\lceil 2^{m}$/10+$\epsilon_{m}\rceil$]
5	2	3	4	4	3	[3,4]
6	5	7	6	8	6	[6,7]
7	11	13	14	13	13	[12,14]
8	23	25	27	27	26	[24,28]
9	49	51	50	55	51	[48,55]
10	99	104	98	107	104	[97,109]
11	199	209	204	204	208	[194,218]

**Table 5:** Number of pairs (u,v) satisfying $\mathrm{Avr}\left(B\left(W^{u}\right)\right)\leq \mathrm{Avr}\left(B\left(W^{v}\right)\right)$ for which 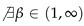 s.t. β(u)≤β(v).

*m*	β	Number of Incompatible Pair of Elements	$2^{m}$
4	(1,1.32]	–	16
5	(1.18,1.22]	–	32
6	1.22	2	64
7	1.22	10	128
8	1.22	36	256
9	1.22	99	512

## Data Availability

The data presented in this study are available within the article.

## Appendix A. Proofs of Results from Section 3

### Appendix A.1. Proof of Proposition 6

**Proposition** (6). *Let f and g be two monomial having the same degree and $x_{h}$ be such that $x_{h}$ and $x_{h}$. Then we have*

(A1) $$f{⪯}_{d}g\;\mathrm{iff}\;x_{h}f{⪯}_{d}x_{h}g.$$

**Proof.** Let $f=x_{i_{1}}\cdots x_{i_{s}}$ and $g=x_{j_{1}}\cdots x_{j_{s}}$ with $f{⪯}_{d}g.$ Also, let $f^{*}=fx_{h}$ and $g^{*}=gx_{h}.$ There are several cases to be examined here:

- If $x_{j_{s}}⪯x_{h}$ or $x_{h}⪯x_{i_{1}}$ then the relation $f^{*}{⪯}_{d}g^{*}$ can easily be verified by using the definition of ${⪯}_{d}.$
- If there is an integer r∈{1,⋯,s} s.t. $x_{i_{r}}⪯x_{h}⪯x_{i_{r+1}}$ and $x_{j_{r}}⪯x_{h}⪯x_{j_{r+1}}$ then the relation $f^{*}{⪯}_{d}g^{*}$ can easily be verified as in the previous step.
- If there are two distinct integers r,t∈{1,⋯,s} s.t. $x_{i_{r}}⪯x_{h}⪯x_{i_{r+1}}$ and $x_{j_{t}}⪯x_{h}⪯x_{j_{t+1}}$ then two cases have to be considered.
  - If t<r then we have $x_{i_{h}}⪯x_{j_{t+1}}⪯\cdots ⪯x_{j_{r}}$ and $x_{i_{t+1}}⪯\cdots ⪯x_{i_{r}}⪯x_{h}$, which implies the following ordering
    (A2) $$x_{i+t+1}⪯\cdots ⪯x_{i_{r}}⪯x_{h}⪯x_{j_{t+1}}⪯\cdots ⪯x_{j_{r}}.$$
    Combining Equation (A2) with the definition of ${⪯}_{d}$ we obtain the desired result, i.e., $f^{*}{⪯}_{d}g^{*}.$
  - t>r then we have $x_{i_{h}}⪯x_{i_{r+1}}⪯\cdots ⪯x_{i_{t}}$ and $x_{j_{r+1}}⪯\cdots ⪯x_{j_{t}}⪯x_{h}$ which implies the following ordering
    (A3) $$x_{j+r+1}⪯\cdots ⪯x_{j_{t}}⪯x_{h}⪯x_{i_{r+1}}⪯\cdots ⪯x_{i_{t}}.$$
    Now, since $x_{h}⪯x_{i_{t}}$ it might be possible to have $j_{s}+\cdots j_{t+1}+h<i_{s}+\cdots +i_{t+1}+i_{t}$, which implies a violation of the partial sum conditions in the definition of ${⪯}_{d}.$ If the next partial sum changes the sign, i.e., $j_{s}+\cdots j_{t+1}+h+j_{t}\geq i_{s}+\cdots +i_{t+1}+i_{t}+i_{t-1}$, by setting $\Delta_{t+1}=\left(j_{s}-i_{s}\right)+\cdots +\left(j_{t+1}-i_{t+1}\right)$ we have the following inequalities
    (A4) $$\Delta_{t+1}+h-i_{t}<0$$
    (A5) $$\Delta_{t+1}+h-i_{t}+j_{t}-i_{t-1}$$
    This implies
    (A6) $$\Delta_{t+1}+h<i_{t}\leq \Delta_{t+1}+h+j_{t}-i_{t-1}.$$
    However, since $j_{t}<i_{t-1}$ the Equation A6) becomes impossible, which completes the proof.

□

### Appendix A.2. Proof of Lemma 2

**Lemma** (2). *Let $f,g\in M_{m},\deg f=\deg g=2$, such that $f{⪯}_{d}g.$ Then $B\left(W_{m}^{f}\right)\leq B\left(W_{m}^{g}\right).$*

**Proof.** Let $f=x_{i_{1}}x_{i_{2}}$ and $g=x_{j_{1}}x_{j_{2}}.$ If gcd(f,g)≠1 then f⪯g, which implies $B\left(W_{m}^{f}\right)\leq B\left(W_{m}^{g}\right).$ Now suppose gcd(f,g)=1, and let $j_{1}-i_{1}\leq j_{2}-i_{2}.$ Denote $\epsilon =j_{1}-i_{1}.$ By definition of ${⪯}_{d}$ and ⪯ we have

(A7) $$f=x_{i_{1}}x_{i_{2}}{⪯}_{d}x_{i_{1}-1}x_{i_{2}+1}{⪯}_{d}\cdots {⪯}_{d}x_{j_{1}}x_{i_{2}+\epsilon }⪯x_{j_{1}}x_{j_{2}}=g.$$

If we prove $x_{i_{1}}x_{i_{2}}{⪯}_{d}x_{i_{1}-1}x_{i_{2}+1}\Rightarrow B\left(W_{m}^{x_{i_{1}}x_{i_{2}}}\right)\leq B\left(W_{m}^{x_{i_{1}-1}x_{i_{2}+1}}\right)$ the proof is finished. By definition, one can easily notice that $B\left(W_{4}^{x_{1}x_{2}}\right)\leq B\left(W_{4}^{x_{0}x_{3}}\right)\Leftrightarrow B\left(W_{m}^{x_{i_{1}}x_{i_{2}}}\right)\leq B\left(W_{m}^{x_{i_{1}-1}x_{i_{2}+1}}\right).$ Hence we are left to prove that $B\left(W_{4}^{x_{1}x_{2}}\right)\leq B\left(W_{4}^{x_{0}x_{3}}\right).$ We have that $B(W_{4}^{x_{0}x_{3}})\left(p\right)=1-{\left(1-{\left(1-{\left(1-p\right)}^{2}\right)}^{4}\right)}^{2}$ and $B(W_{4}^{x_{1}x_{2}})\left(p\right)={\left(1-{\left(1-p^{2}\right)}^{4}\right)}^{2}.$ By writing the two polynomials in the Bernstein basis, and using Theorem 4 we have $B\left(W_{4}^{x_{1}x_{2}}\right)=\mathrm{Rel}\left(C^{\left(0,1,1,0\right)}\right)$ and $B\left(W_{4}^{x_{0}x_{3}}\right)=\mathrm{Rel}\left(C^{\left(1,0,0,1\right)}\right)$ with

$$\begin{array}{cc} N_{i}\left(C^{\left(0,1,1,0\right)}\right) & =0,0,0,0,16,192,1008,3040,5828,7456,6552,4048,1788,560,120,16,1\\ N_{i}\left(C^{\left(1,0,0,1\right)}\right) & =0,0,0,0,32,320,1456,3984,7042,8400,7000,4176,1804,560,120,16,1 \end{array}$$

As $N_{i}\left(C^{\left(0,1,1,0\right)}\right)\leq N_{i}\left(C^{\left(1,0,0,1\right)}\right)$ for all i∈{0,⋯,16} we conclude the proof. □

### Appendix A.3. Proof of Theorem 5

**Theorem** (5). *Polar codes over the Binary Erasure Channel are strongly decreasing monomial codes.*

**Proof.** The proof is based on two induction steps. First the parameter *m* is fixed and we prove that the result holds for any 1≤s≤m. Secondly we use induction on *m*. Firstly, fix *m* and use an induction argument on the degree of monomial, namely on s. We also suppose that gcd(f,g)=1. For s=1 we have that $⪯={⪯}_{d}$ so the result is obvious. For s=2 use Lemma 2. Now suppose that for any $f{⪯}_{d}g$ with degf=degg=s−1 we have that $B\left(W_{m}^{f}\right)\leq B\left(W_{m}^{g}\right).$ Let $f=x_{i_{1}}\cdots x_{i_{s}}$ and $g=x_{j_{1}}\cdots x_{j_{s}}$ such that $g{⪯}_{d}f$ with the usual convention $i_{1}<\cdots <i_{s}$ and $j_{1}<\cdots <j_{s}$. Then we have two cases. Either if $f/x_{i_{s}}{⪯}_{d}g/x_{j_{s}}$ or if $x_{i_{1}}\leq x_{j_{1}}$ then we have $B\left(W_{m}^{f}\right)\leq B\left(W_{m}^{g}\right)$. Indeed, in the first case we have that

$$\begin{array}{cc} B\left(W_{m}^{f}\right) & =B\left({\left(W_{m-j_{s-1}-1}^{x_{i_{s}}}\right)}_{j_{s-1}+1}^{f/x_{i_{s}}}\right)\leq B\left({\left(W_{m-j_{s-1}-1}^{x_{j_{s}}}\right)}_{j_{s-1}+1}^{f/x_{i_{s}}}\right)\\ \; & \leq B\left({\left(W_{m-j_{s-1}-1}^{x_{j_{s}}}\right)}_{j_{s-1}+1}^{g/x_{j_{s}}}\right)=B\left(W_{m}^{g}\right). \end{array}$$

In the second case when $x_{i_{1}}\leq x_{j_{1}}$ the proof works in the same way. If we are not in the previous case it means that $j_{1}<i_{1}$ and $i_{s}<j_{s}.$ We know that there is l∈{1,⋯,s−1} for which $i_{l}>j_{l}.$ First we treat the two extreme cases l=1 or l=s−1. If l=1 this implies that $j_{k}\geq i_{k}$ for all k>1. Let $\delta =\min\{j_{2}-i_{2},i_{1}-j_{1}\}.$ Then

$$f=x_{i_{1}}x_{i_{2}}\cdots x_{i_{s}}{⪯}_{d}x_{i_{1}-\delta }x_{i_{2}+\delta }x_{i_{3}}\cdots x_{i_{s}}{⪯}_{d}x_{j_{1}}\cdots x_{j_{s}}=g.$$

In this case, either $x_{i_{1}-\delta }=x_{j_{1}}$ or $x_{i_{2}+\delta }=x_{j_{2}}.$ Hence, by Lemma 2 and using the order relation ⪯ we obtain

$$\begin{array}{cc} B\left(W_{m}^{f}\right) & =B\left({\left(W_{m-j_{2}-1}^{f/(x_{i_{1}}x_{i_{2}})}\right)}_{j_{2}+1}^{x_{i_{2}}x_{i_{1}}}\right)\leq B\left({\left(W_{m-j_{2}-1}^{f/(x_{i_{1}}x_{i_{2}})}\right)}_{j_{2}+1}^{x_{i_{2}+\delta }x_{i_{1}-\delta }}\right)\\ \; & \leq B\left({\left(W_{m-j_{2}-1}^{x_{i_{s}}\cdots x_{i_{3}}}\right)}_{j_{2}+1}^{x_{j_{2}}x_{j_{1}}}\right)\leq B\left({\left(W_{m-j_{2}-1}^{x_{j_{s}}\cdots x_{j_{3}}}\right)}_{j_{2}+1}^{x_{j_{2}}x_{j_{1}}}\right)=B\left(W_{m}^{g}\right). \end{array}$$

If l=s−1, by putting $\delta =i_{s-1}-j_{s-1}$ and taking into account that $\delta \leq j_{s}-i_{s}$ we obtain

$$\begin{array}{cc} B\left(W_{m}^{f}\right) & =B\left({\left(W_{m-j_{s-2}-1}^{x_{i_{s}}x_{i_{s-1}})}\right)}_{j_{s-2}+1}^{f/(x_{i_{s}}x_{i_{s-1}})}\right)\leq B\left({\left(W_{m-j_{s-2}-1}^{x_{i_{s}+\delta }x_{i_{s-1}-\delta })}\right)}_{j_{s-2}+1}^{f/(x_{i_{s}}x_{i_{s-1}})}\right)\\ \; & =B\left({\left(W_{m-j_{s-2}-1}^{x_{i_{s}+\delta }x_{j_{s-1}})}\right)}_{j_{s-2}+1}^{x_{i_{s-2}}\cdots x_{i_{1}}}\right)\leq B\left({\left(W_{m-j_{s-2}-1}^{x_{j_{s}}x_{j_{s-1}})}\right)}_{j_{s-2}+1}^{x_{j_{s-2}}\cdots x_{j_{1}}}\right)=B\left(W_{m}^{g}\right). \end{array}$$

When 1<l<s−1 suppose that $i_{s}-j_{l}<j_{l+1}-i_{s}$ and denote by $\delta_{l,s}=i_{s}-j_{l}.$ Using the definition of ${⪯}_{d}$ we have

$$h=x_{j_{1}}\cdots x_{j_{l}+\delta_{l,s}}x_{j_{l+1}-\delta_{l,s}}\cdots x_{j_{s}}{⪯}_{d}\cdots {⪯}_{d}x_{j_{1}}\cdots x_{j_{l}+1}x_{j_{l+1}-1}\cdots x_{j_{s}}{⪯}_{d}x_{j_{1}}\cdots x_{j_{s}}=g.$$

Notice that $h=x_{j_{1}}\cdots x_{j_{l-1}}x_{i_{s}}x_{j_{l+1}-i_{s}+j_{l}}\cdots x_{j_{s}}.$ Next, we prove that $f{⪯}_{d}h.$ Since $\gcd\left(f,h\right)=x_{i_{s}}$, we can use Lemma 6 and demonstrate $f/x_{i_{s}}{⪯}_{d}h/x_{i_{s}}$, i.e., $x_{i_{1}}\cdots x_{i_{s-1}}{⪯}_{d}x_{j_{1}}\cdots x_{j_{l-1}}x_{j_{l+1}-i_{s}+j_{l}}x_{j_{l+2}}\cdots x_{j_{s}}.$ As,

(A8) $$x_{i_{l+1}}⪯\cdots ⪯x_{i_{s}}⪯x_{j_{l+1}}⪯\cdots ⪯x_{j_{s}},$$

we obtain $x_{i_{l+1}}\cdots x_{i_{s-1}}{⪯}_{d}x_{j_{l+2}}\cdots x_{j_{s}}$, simply by verifying

(A9) $$\forall \;t\in \left\{0,\cdots ,s-l-2\right\}\;\sum_{k=0}^{t}i_{s-1-k}\leq \sum_{k=0}^{t}j_{s-k}.$$

The next partial sums inequalities,

(A10) $$\left(i_{k}+\cdots +i_{l-1}\right)+i_{l}+i_{l+1}+\cdots +i_{s-1}<\left(j_{k}+\cdots +j_{l-1}\right)+j_{l+1}-i_{s}+j_{l}+j_{l+2}+\cdots +j_{s}$$

are verified from the relation $f{⪯}_{d}g.$ So we check the partial sums step by step:

1. $i_{s-1}<i_{s}<j_{l+1}<j_{s}$ and thus $x_{i_{s-1}}{⪯}_{d}x_{j_{s}}$
2. $i_{s-1}+i_{s-1}<j_{s-1}+j_{s}$ and thus $x_{i_{s}-2}x_{i_{s-1}}{⪯}_{d}x_{j_{s-1}}x_{j_{s}}$
3. ⋯
4. $i_{l+1}+\cdots +i_{s-1}<j_{l+2}+\cdots +j_{s}$ and thus $x_{i_{l+1}}\cdots x_{i_{s-1}}{⪯}_{d}x_{j_{l+2}}\cdots x_{j_{s}}$
5. $i_{l}+i_{l+1}+\cdots +i_{s-1}<j_{l+1}-i_{s}+j_{l}+j_{l+2}+\cdots +j_{s}$ by definition of $f{⪯}_{d}g$ and thus $x_{i_{l}}\cdots x_{i_{s-1}}{⪯}_{d}x_{j_{l+1}-i_{s}+j_{l}}\cdots x_{j_{s}}$
6. ⋯

Hence we have that $f/x_{i_{s}}{⪯}_{d}h/x_{i_{s}}$ which implies, using the induction hypothesis, that $B\left(W_{m}^{f/x_{i_{s}}}\right)\leq B\left(W_{m}^{h/x_{i_{s}}}\right),$ from which we deduce $B\left(W_{m}^{f}\right)\leq B\left(W_{m}^{h}\right).$ Also,

$$\begin{array}{cc} B\left(W_{m}^{h}\right) & =B\left({\left({\left(W_{}^{x_{j_{s}}\cdots x_{j_{l+2}}}\right)}^{x_{j_{l+1-\delta_{l,s}}}x_{j_{l+\delta_{l,s}}}}\right)}^{x_{j_{l-1}}\cdots x_{j_{1}}}\right)\\ \; & =B\left({\left({\left(W^{*}\right)}^{x_{j_{l+1-\delta_{l,s}}}x_{j_{l+\delta_{l,s}}}}\right)}^{x_{j_{l-1}}\cdots x_{j_{1}}}\right)\leq B\left({\left({\left(W^{*}\right)}^{x_{j_{l+1}}x_{j_{l}}}\right)}^{x_{j_{l-1}}\cdots x_{j_{1}}}\right)\\ \; & =B\left({\left({\left(W_{}^{x_{j_{s}}\cdots x_{j_{l+2}}}\right)}^{x_{j_{l+1}}x_{j_{l}}}\right)}^{x_{j_{l-1}}\cdots x_{j_{1}}}\right)=B\left(W_{m}^{g}\right). \end{array}$$

Secondly, we use induction on the number of variables m. For the first values of *m*, i.e., m≤4 it is straightforward to check the result. Let $f=x_{i_{1}}\cdots x_{i_{s}}$ and $g=x_{j_{1}}\cdots x_{j_{s}}$ such that $g{⪯}_{d}f$ with the usual convention $i_{1}<\cdots <i_{s}$ and $j_{1}<\cdots <j_{s}$. The following cases are possible

- If $i_{s}=j_{s}=m$ then we have $W_{m+1}^{f}={\left(W_{1}^{f_{m}}\right)}_{m}^{f_{\left[0,m-1\right]}}$ and $W_{m+1}^{g}={\left(W_{1}^{g_{m}}\right)}_{m}^{g_{\left[0,m-1\right]}}.$ Since $f_{\left[0,m-1\right]}{⪯}_{d}g_{\left[0,m-1\right]}$ we have by the induction hypothesis
  (A11) $$B\left({\left(W_{1}^{x_{m}}\right)}_{m}^{f_{\left[0,m-1\right]}}\right)\leq B\left({\left(W_{1}^{x_{m}}\right)}_{m}^{g_{\left[0,m-1\right]}}\right).$$
- Else, by the definition of the order we necessary have $j_{s}>i_{s}.$ We also have that
  $$h=x_{j_{1}}\cdots x_{j_{s-1}+1}x_{j_{s}-1}{⪯}_{d}x_{j_{1}}\cdots x_{j_{s-1}}x_{j_{s}}=g.$$
  Which implies that $B\left(W_{m+1}^{h}\right)\leq B\left(W_{m+1}^{g}\right).$ In the same time notice that $f{⪯}_{d}h$ and ind(f),ind(h)∈{0,⋯,m−1}. Therefore we obtain
  (A12) $$B\left(W_{m+1}^{f}\right)\leq B\left(W_{m+1}^{h}\right)\leq B\left(W_{m+1}^{g}\right).$$

□
